# A Pleasant Visit

**Published:** 1891-05

**Authors:** 


### A Pleasant Visit.

On the 15th of March, 1891, the next day after the adjournment of the Mississippi Valley Dental Association, several members, namely, Doctors James Leslie, A. O. Hise, J. J. R. Patrick, E. G. Betty, L. E. Custer, J. Taft and William Taft, after due preparation, made their way to Xenia, Ohio, to call upon that veteran member of the profession, Dr. George Watt.

It is, of course, known to all of the profsssion that the Doctor has been an invalid for many years, often suffering very greatly, but on this occasion he was found really better than was expected. He received the party with seeming delight, and entertained them for the day in a way most highly satisfactory to the visitors. Though the Doctor's physical strength is much impaired, it is remarkable to what degree his mental faculties retain their strength and power, they seemingly would be about equal for the old time work if the physical strength was present. The intellect is strong and willing, but the body is weak, and though he has been practically out of the profession for many years, yet, he is interested in, and even enthusiastic, in whatever pertains to the interest of dentistry. He has, under the circumstances, kept up remarkably well with its literature. It was a matter of gratification to every one of these visitors that they could enjoy a full day with the Doctor and have much of the old time pleasant conversation and repartee. May he remain to enjoy many more such pleasant occasions, is the wish of all these visitors.

---

"Oh, yes! Oh, yes!" We've got 'em this time, the largest, rarest and greatest symposeum of unique attractions ever gathered under one case—roof. "No flies any more, everybody invited, and don't you forget it" May 13th and 14th; come and see Miller, Shepard, Pierce, Kirk and a host of others, each in a great act, "*Don't forget the date.*" "One special feature at the enormous expense of $300, will pay any one for coming." "The eyes of the world are upon us"—President. Does this remind you of anything?—H.

